# Root Architecture Diversity and Meristem Dynamics in Different Populations of *Arabidopsis thaliana*

**DOI:** 10.3389/fpls.2016.00858

**Published:** 2016-06-16

**Authors:** Pamela Aceves-García, Elena R. Álvarez-Buylla, Adriana Garay-Arroyo, Berenice García-Ponce, Rodrigo Muñoz, María de la Paz Sánchez

**Affiliations:** ^1^Laboratorio de Genética Molecular, Epigenética, Desarrollo y Evolución de Plantas, Instituto de Ecología, Universidad Nacional Autónoma de México, MéxicoMexico; ^2^Departamento de Ecología y Recursos Naturales, Facultad de Ciencias, Universidad Nacional Autónoma de México, MéxicoMexico

**Keywords:** natural variation, root morphology, RAM, stem cell niche, *Arabidopsis* accessions

## Abstract

*Arabidopsis thaliana* has been an excellent model system for molecular genetic approaches to development and physiology. More recently, the potential of studying various accessions collected from diverse habitats has been started to exploit. Col-0 has been the best-studied accession but we now know that several traits show significant divergences among them. In this work, we focused in the root that has become a key system for development. We studied root architecture and growth dynamics of 12 *Arabidopsis* accessions. Our data reveal a wide variability in root architecture and root length among accessions. We also found variability in the root apical meristem (RAM), explained mainly by cell size at the RAM transition domain and possibly by peculiar forms of organization at the stem cell niche in some accessions. Contrary to Col-0 reports, in some accessions the RAM size not always explains the variations in the root length; indicating that elongated cell size could be more relevant in the determination of root length than the RAM size itself. This study contributes to investigations dealing with understanding the molecular and cellular basis of phenotypic variation, the role of plasticity on adaptation, and the developmental mechanisms that may restrict phenotypic variation in response to contrasting environmental conditions.

## Introduction

Natural variation is the main source for evolutionary change and the substrate for selection and adaptation of populations to a specific environment (Alonso-Blanco et al., 2009; Hancock et al., 2011; Agren and Schemske, 2012; Richards et al., 2012). Although great interest has been devoted to study genetic variation, we still have a sketchy understanding of the molecular basis and constraints of phenotypical variation. Plants are sessile organisms that require and rely on a wide range of plastic responses, that are underlined by complex genetic and epigenetic mechanisms and which enable their adjustment to the changing environment that they encounter during their life-cycles (Falke et al., 2013; Eichten et al., 2014). *Arabidopsis thaliana* (*Arabidopsis* here after) populations that have been collected from particular geographic locations are commonly referred to accessions; these varieties show an ample range of variation in their phenotypical traits (Assmann, 2013; Aliniaeifard and van Meeteren, 2014; Ristova and Busch, 2014). They comprise a key resource to understand the molecular basis of variation, the role of plasticity in adaptive evolution, as well as the constructive evolutionary role of the environment (Mitchell-Olds and Schmitt, 2006; Fusco and Minelli, 2010).

*Arabidopsis* accessions have been used as “natural mutants” to assess the function of individual genes and the specific genotype-environment relationship. Contrary to mutant approaches, such accessions eliminate the use of T-DNA, mutagens, or RNA interferences that could be affecting other physiological processes. This approach can yield biological significant gene function information (Alonso-Blanco et al., 2009). For instance, the function of FRIGIDA and FLOWERING LOCUS C, two proteins involved in the networks underlying flowering time in *Arabidopsis* was elucidated based on their variation in different accessions (Koornneef et al., 1994; Johanson et al., 2000). While most of such studies have concerned aerial phenotypic variation, root natural variation has also been described in fewer cases (Beemster et al., 2002; Mouchel et al., 2004; Ristova and Busch, 2014). The root system is fundamental for nutrient, minerals and water uptake, as well as plant support (Aiken and Smucker, 1996; Pacheco-Villalobos and Hardtke, 2012). Thus, root development, architecture and morphology can be affected also by environmental factors, such as nutrient availability, humidity and temperature to confer adaptive advantages or resistances under some environmental conditions and some of them have been fixed during evolution (Ristova and Busch, 2014). However, we still do not understand the molecular genetics and developmental basis of relevant root traits, their plasticity and role, in conjunction with such environmental factors, during adaptive evolution.

The *Arabidopsis* root is a radial and symmetric organ, comprised of concentric files of different cell types that from the outside to the inside of the organ are: epidermis, cortex, endodermis, pericycle and vascular tissues. Along the longitudinal axis the primary root, has three different zones: at the tip of the root is the root apical meristem (RAM), which is conformed by the stem cell niche that comprise of a group of lower mitotic activity cells, called quiescent center (QC) surrounded by four types of stem or initial cells (epidermis/lateral root cap, cortex/endodermal, vascular/pericycle and columella). These stem cells divide asymmetrically to give rise to self-renewing stem cells and daughter cells that in turn divide several times to form the epidermis and lateral root cap cells, cortex and endodermal cells, stele as well as columella cells (Dolan et al., 1993). The meristematic zone contains the proliferation domain, where cells attain the highest proliferation rate and the transition domain with cells that start to elongate but have not yet started rapid elongation and still proliferate (Ivanov and Dubrovsky, 2013). After the RAM, cells enter a zone of rapid elongation at the Elongation Zone (EZ) and ultimately acquire their final characteristics at the Maturation Zone (MZ). While the radial root organization is rather canalized, the longitudinal one is more plastic in response to various environmental conditions.

The root system architecture consists also of the three dimensional distribution of the primary and lateral roots (Lynch, 1995), which can be altered by environmental conditions (Malamy, 2005). Nutrient availability and environmental conditions can alter cell number, length, angle, and growth rate of roots as well as lateral root emergence and root hair density (Malamy, 2005; Ristova and Busch, 2014). Root system may grow toward more favorable conditions, activating different types of tropisms (Zhang and Forde, 1998; Linkohr et al., 2002; Remans et al., 2006; Giehl et al., 2012). Positive gravitropism and hydrotropism along with negative phototropism play important roles in root system architecture; while primary roots grow along the gravity vector, lateral roots initially grow in a more horizontal orientation and only later display positive gravitropism (Kiss et al., 2002). Roots also grow in response to moisture gradients by positive hydrotropism to acquisition of water (Moriwaki et al., 2013) and ensure proper root growth into the soil where nutrients and water are available.

*Arabidopsis* accessions that were collected originally from different geographical areas show variation in its root system architecture presumably associated with adaptation to the prevailing environmental conditions (Beemster et al., 2002). Several reports have been focused on analyzing *Arabidopsis* root natural variation in response to different growth conditions (Ristova and Busch, 2014); however, little is known about the variation of the RAM and the stem cell niche morphology. To analyze such natural variation in *Arabidopsis*, we studied 12 different accessions that originally were obtained from contrasting habitats and we compared their root system architecture, their root growing dynamics and the RAM size, as well as the stem cell niche morphology. We also analyzed root size variation with respect to environmental conditions for measure the adaptive value of root development through putative selective agents, as precipitation and temperature. Our data show a wide variability in the root architecture and in the root size among accessions, the latter positively correlates with annual precipitations of place of collection. Moreover, RAM length is also variable among accession. Such variation seems to be explained by RAM cell size at the transition domain and possibly by tissue organization at the stem cell niche in some accessions. In addition, contrary to reports that indicate that high proliferation rate at the RAM produces more cells that are able to differentiate and expand, resulting in a high root length; we found that in some accessions, root length is not directly associated with meristem size, therefore elongated cell size could be more important in the determination of root length than the RAM size itself. We think that the differences in root architecture are of special interest to understand the capacity of plant natural adaptation to a particular environment and for future search of genetic and epigenetic mechanisms involved under a specific genotype-environment contexts underlying root architecture and morphology natural variation.

## Materials and Methods

### *Arabidopsis* Accessions

The *Arabidopsis thaliana* accessions used in this work (**Table 1**) were: Bay-0 (N954), CIBC10 (N22229), Cvi-0 (N1096), Fr-2 (N1168), HR5 (N22205), Ove-0 (N1434), Se-0 (N1502), Sha (N6180), and Ta-0 (N1548) from Nottingham *Arabidopsis* Stock Centre (NASC), whereas Col-0, L*er* and Ws are the common accessions used in our laboratory. The geographical coordinates, latitude and longitude were obtained from the 1001 genome database of *Arabidopsis* available at http://signal.salk.edu/atg1001/3.0/gebrowser.php (Weigel and Mott, 2009). The average annual temperature and average annual precipitation were calculated using the AQUASTAT database from FAO 2015 available at http://www.fao.org/nr/water/aquastat/quickwms/climate.htm

**Table 1:** Geographic distribution of the *Arabidopsis thaliana* accessions.

Accession	NASC stock number	Location	Latitude	Longitude	Altitude (m)	Average annual temperature (°C)	Average annual precipitation (mm)
Col-0	–	Columbia, EUA	38.50	-92.50	50	12.85	82.25
Bay-0	N954	Bayreuth, Germany	49.00	11	360	7.72	65.83
CIBC10	N22229	Ascot/Berks, UK	51.41	-0.64	Unknown	9.75	58
Cvi-0	N1096	Cape Verdi Islands	15.11	-23.62	1200	22.58	18.25
Fr-2	N1168	Frankfurt, Germany	50.00	8.50	100	9.68	51.08
HR5	N22205	Ascot/Berks, UK	51.40	-0.64	Unknown	9.76	57.98
L*er*	–	Landsberg, Germany	47.98	10.87	50	7.88	83.38
Ove-0	N1434	Ovelgoenne, Germany	53.50	8.50	50	8.86	63.75
Se-0	N1502	San Eleno, Spain	41.58	2.50	100	14.35	62.97
Sha	N6180	Pamiro-Alay, Tadjikistan	39.00	72.00	3400	-9.54	90.58
Ta-0	N1548	Tabor, Czech republic	49.50	14.50	450	7.45	50.25
Ws	–	Wassilewskija, Russia	52.30	30	Unknown	6.82	50.83

### Plant Growth Conditions

Seeds of the 12 *Arabidopsis* accessions used in this study were disinfected in 20% sodium hypochlorite and 0.01% of Tween-20 for 15 min. Then seeds were stratified at 4°C for 5 days under dark conditions and sown on square petri dishes containing 0.2X Murashige and Skoog salts (MP Biomedicals), 0.05% MES (SigmaAldrich), 1% sucrose (SigmaAldrich) and 1% agar (Becton, Dickinson and Company) at pH = 5.6. The plates were vertically incubated in a growth chamber at 22°C under long-day (LD; 16 h light/8 h dark) conditions with a light intensity of 110 μEm^-2^ s^-1^.

### Morphometric Analysis

**FIGURE 1:**
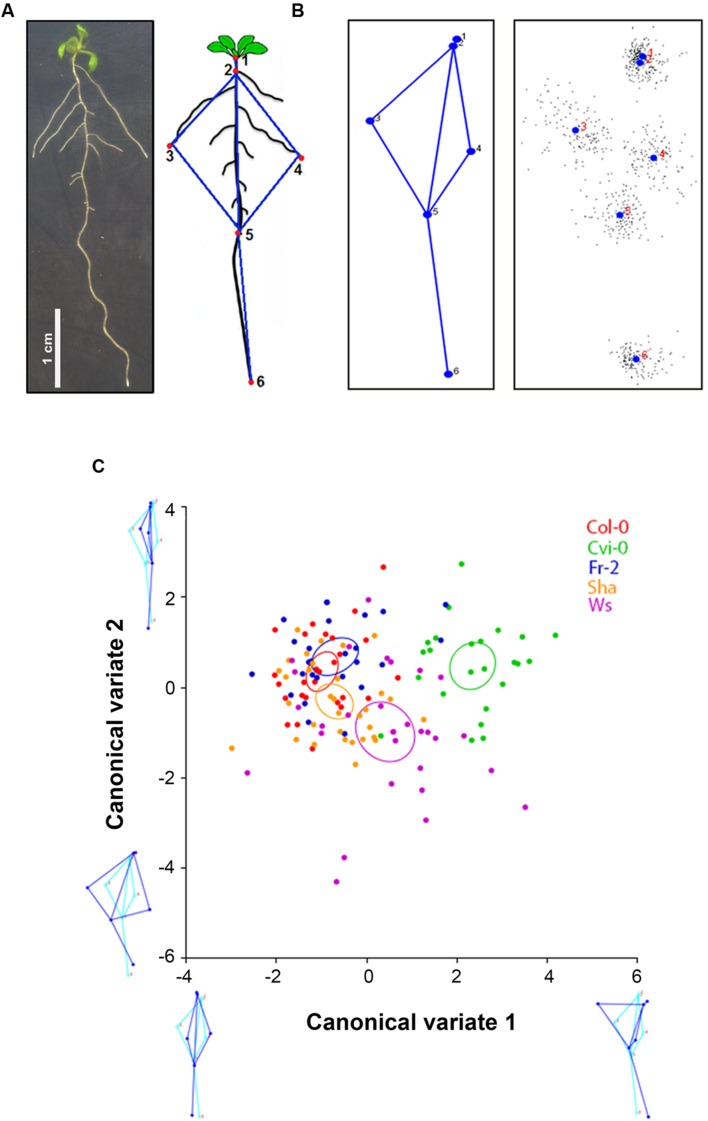
***Arabidopsis* root morphometric variation. (A)** *Arabidopsis* root morphology and the landmarks used for morphometric analysis: landmark 1, position on the transition between shoot and root; landmark 2, the initiation of first lateral root, landmarks 3 and 4, the tips of longest lateral roots in each side; landmarks 5 and 6, the initiation of the latest lateral root emerged and the apex of the primary root, respectively. **(B)** Root average shape of all *Arabidopsis* accessions, the black dots show the variation of each population. **(C)** Canonical variate analysis of Col-0, Cvi-0, Fr-2, Sha and Ws root shape variations. The plot shows the distribution of accession root shapes and the color ellipses display the accession average shapes with 95% confidence intervals. The diagram of root average shape is displayed in light blue and the contrasting shapes of roots are shown in dark blue (*n* > 25), statistical significance at *P* < 0.05.

For morphometric assays, square dishes with seedlings of 9 days after germination (dag) were scanned at 1200 dpi. Landmarks were drawn in each seedling image (**Figure 1A**) using TpsDig2 and TpsUtil software (Rohlf, 2010) and the least-squares procrustes superimpositions were made using MorphoJ software (Klingenberg, 2011), which also was used for analysis of canonical variables (CVA) and principal component analysis (PCA).

### Kinematic Analysis

Growth rate and root length of each accession were obtained by marking the position of the root tip every 24 h on the back of plates. At 10 dag plates were digitized and root length was measured using Fiji software (Schindelin et al., 2012).

### Microscopy Analysis

For light microscopy, seedlings were cleared with 80% chloral hydrate and 10% glycerol and starch granules of the columella cells were stained with 1% lugol (SigmaAldrich). Root tissues were visualized using Nomarski optics under an Olympus BX60 microscope with a dry 40X objective, and photographed with an Evolution MP COLOR of Media Cybernetics camera. For confocal, roots were stained with propidium iodide (PI) solution (10 μg/mL) and images were acquired using Olympus FV1000 confocal microscope with a oil immersion 40X objective at 543/560nm excitation and emission laser. RAM length and the number or length of cortex cells in the proliferation domain from the QC cells to the first elongated cortex cell (Casamitjana-Martinez et al., 2003; Hacham et al., 2011) were obtained using Fiji software (Schindelin et al., 2012) and using plants of 4 and 5 dag because the meristem is more easy of viewing at this age.

### Statistical Analysis

Quantitative morphometric analyses were performed using the MorphoJ software (Klingenberg, 2011) and ANOVA test. For the kinematic analysis, the statistical significance was obtained by ANOVA with *post hoc* Tukey tests using the PRISMA software (Moher et al., 2009). R software (R Development Core Team, 2015) was used to estimate the correlation between root length and environmental conditions using non-parametric Spearman test. Pearson’s correlation tests were obtained to assess if root growth rate and root length show a statistically significant association.

To analyze the differences in cell length among accessions (**Figure 7B**), the data (five meristematic cells before the first elongated cell) was log normalized and a two-way analysis of variance (ANOVA) was performed. The first factor measured was the effect of meristematic cell position (from first to fifth position) on cell length; the second was the effect of belonging to each accession and third was the interaction between the two factors.

## Results

### Root Morphometric Analyses of the *Arabidopsis* Accessions

*Arabidopsis* has been collected from a wide range of habitats distributed mainly over the Northern hemisphere, thus each accession has acquired some specific characteristics in response to environmental conditions. For this work, we selected 12 accessions from different habitats and regions with contrasting environmental conditions, including accessions from America (Columbia, Col-0), Asia (Pamiro-Alay, Sha; and Wassilewskija, Ws), Africa (Cape Verdi Islands, Cvi-0) and Europe (San Eleno, Se-0; Bayreuth, Bay; Frankfurt, Fr-2; Ovelgoenne, Ove-0; Landsberg *erecta*, L*er*; Ascot/Berks, CIBC10 and HR5; and Tabor, Ta-0) (**Table 1**). Their root phenotypes were studied under our plant growth conditions. We found a wide range of root architectures and variation in other root traits as well (Supplementary Figure S1).

Quantitative analyses of root system architecture of the *Arabidopsis* accessions used were obtained with geometric morphometrics. For this, we defined six landmarks (**Figure 1A**), which cover the root architecture including: a position on the transition between shoot and root (landmark 1), the initiation of the first lateral root (landmark 2), the tip of the longest lateral roots in each side (landmark 3 and 4), the initiation of the latest lateral emerged root (landmark 5) and the apex of the primary root (landmark 6). According to these landmarks we obtained the morphological pattern, in where all accessions fit (**Figure 1B**). To eliminate variation in size, position and orientation of each accession, and thereby obtain the coordinates superimposed landmarks, that are the core of geometric morphometric for multivariate analysis (Klingenberg, 2011), we performed the least-squares procrustes superimpositions (Dryden and Mardia, 1998) to fitting a model that completely describes the differences between *Arabidopsis* root shapes of each accession to fit a very simple model that only takes into consideration global parameters such as differences in rotation, translation and scale. Each *Arabidopsis* accession was pairwise-compared, except for HR5, because it has very few and short lateral roots, which do not fit in the landmarks coordinates (Supplementary Figure S1). According to procrustes distances obtained in these analysis (Supplementary Table S1), we selected accessions with higher (Cvi-0 and Ws), intermediate (Sha) and lower (Fr-2 and Col-0) procrustes distances to identify the morphometric patterns from each *Arabidopsis* accession and its differences in the multivariate state-space by analysis of CVA. This parameter maximizes the differences among accessions relative to the variation among them, identifying groups according to their morphology. The scatter plot shows a similar distribution among accessions, although the accession average shapes (*P* < 0.05) were clearly distinct for Cvi-0 and Ws, that form two different clusters which are separated from Col-0, Fr2 and Sha overlapping clusters, being Sha accession the most distal one (**Figure 1C**). The score of CV1 (first axis) indicates that root morphology differs mainly in the vertical displacement of the first longest left lateral roots, whereas CV2 (second axis) was associated to differences in the orientation of lateral roots, which are closer to primary root, and the displacement of the last lateral root (**Figure 1C**).

**FIGURE 2:**
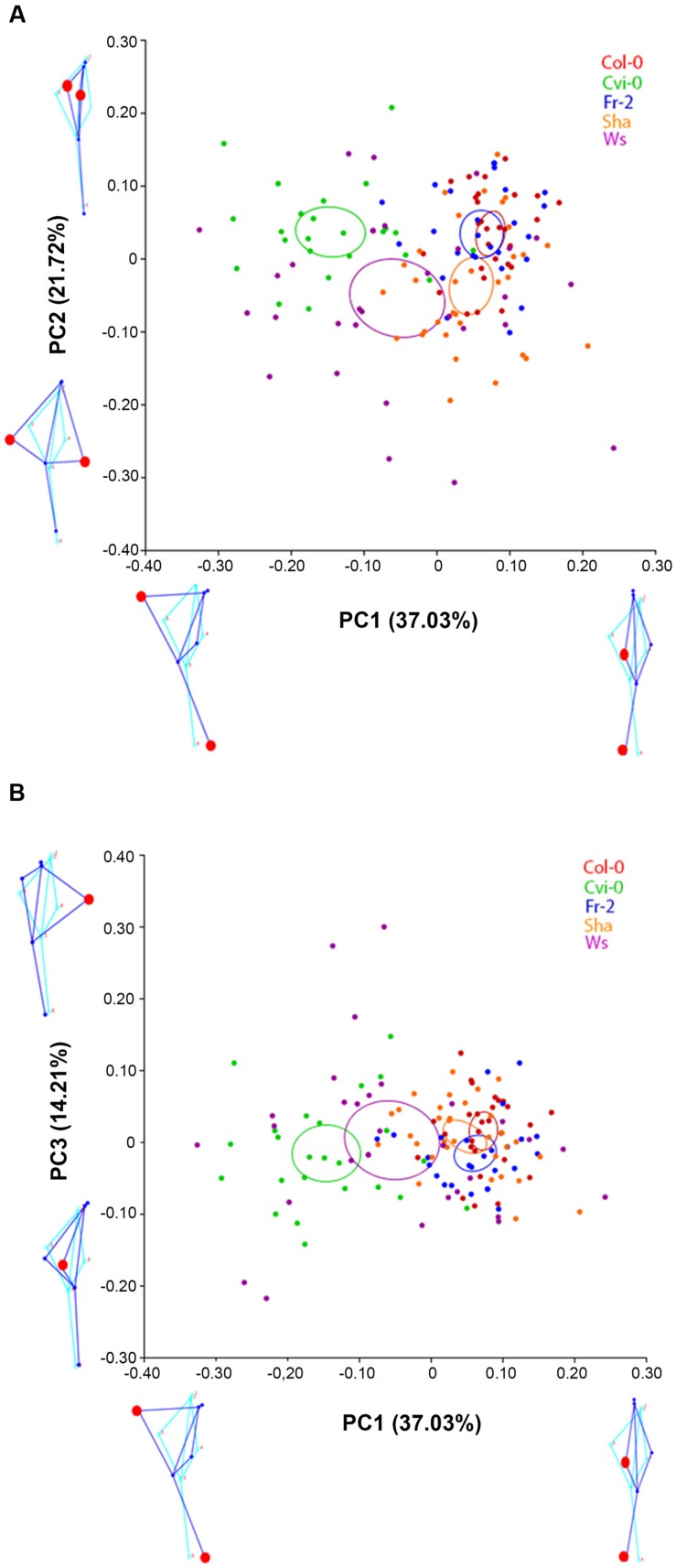
**Principal component analysis of *Arabidopsis* root shapes.** Principal component analysis for Col-0, Cvi-0, Fr-2, Sha and Ws accessions: **(A)** Plot of PC1 vs. PC2 and **(B)** Plot of PC1 vs. PC3. The color ellipses display the accession average shapes with 95% confidence. The diagram of root average shape is displayed in light blue and the contrasting shapes of root are shown in dark blue (*n* > 25), statistical significance at *P* < 0.05.

**FIGURE 3:**
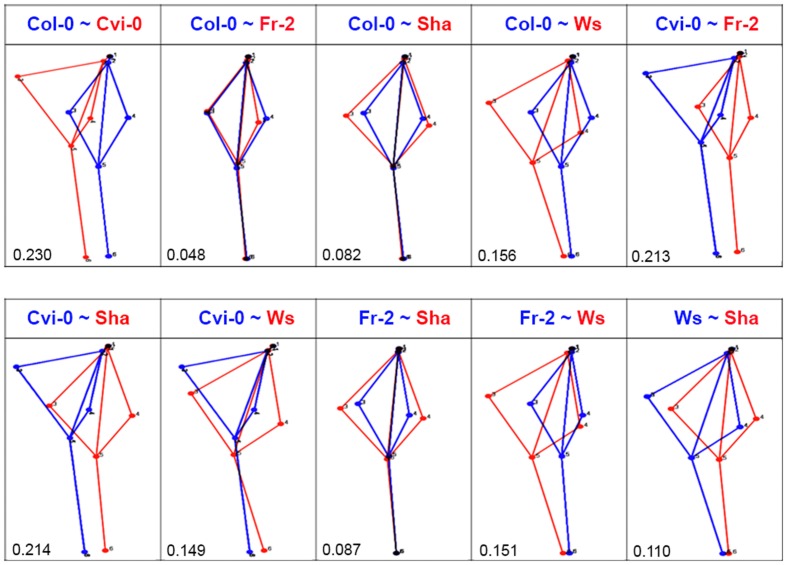
**Comparative analysis of root shape variations.** The pairwise comparisons between *Arabidopsis* accession shapes that are represented in blue or red as indicated in each case (*n* > 20). The procrustes distance in each comparison is shown below. Value closer to 0 means morphology superimposition or high similarity.

To display the main patterns of variation in shape space, we used global PCA (Klingenberg et al., 1996). In this analysis we identified that the first three principal components (PCA1 to 3) covered over 72.96% of variation of root morphology among *Arabidopsis* accessions. PCA1 accounted for 37.03% of the observed variation, whereas PCA2 and PCA3 accounted for 21.72% and 14.21%, respectively (**Figure 2**). Using eigenvalues higher than 0.3 (ter Braak, 1988), we identified that the landmarks associated to main variation of PCA1 were the distance and orientation between the longest left lateral root and the primary root, as well as the orientation of the primary root, while for PCA2 the landmarks were the distance between the longest right and left lateral roots and primary root (**Figure 2A**). The landmarks for PCA 3 were the orientation between the longest right lateral root and the primary root (**Figure 2B**). The morphology variations for PCA1 were more evident in the pairwise comparisons between contrasting shapes, like Cvi-0 with Col-0, Fr-2 or Ws (**Figure 3**), whereas for PCA2 were more evident between Ws with Fr-2, and Col-0 or Cvi-0 with Fr-2 (**Figure 3**).

### *Arabidopsis* Root Length Variation and Its Relationship to Environmental Conditions

**FIGURE 4:**
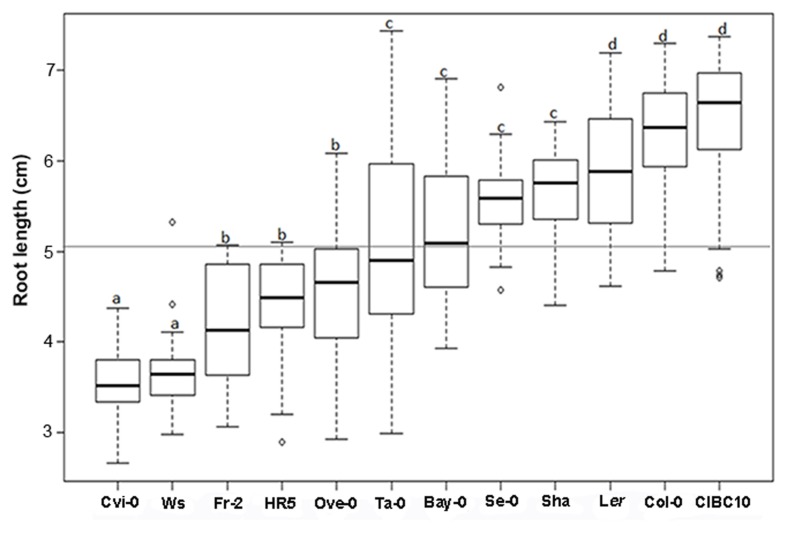
***Arabidopsis* root length variations.** Box plot for root length size in centimeters (cm) from Cvi-0, Ws, Fr-2, HR5, Ove-0, Ta-0, Bay-0, Se-0, Sha, L*er*, Col-0 and CIBC10 at 9 dag. Dark horizontal lines represent the median, with the box representing the 25th and 75th percentiles, the whiskers the 1.5 IQR limits and outliers are represented by dots for each *Arabidopsis* accession (*n* > 30). The gray line represents the root length mean for all accessions and the letters indicate significant differences at *P* < 0.001 among accessions by ANOVA test.

**FIGURE 5:**
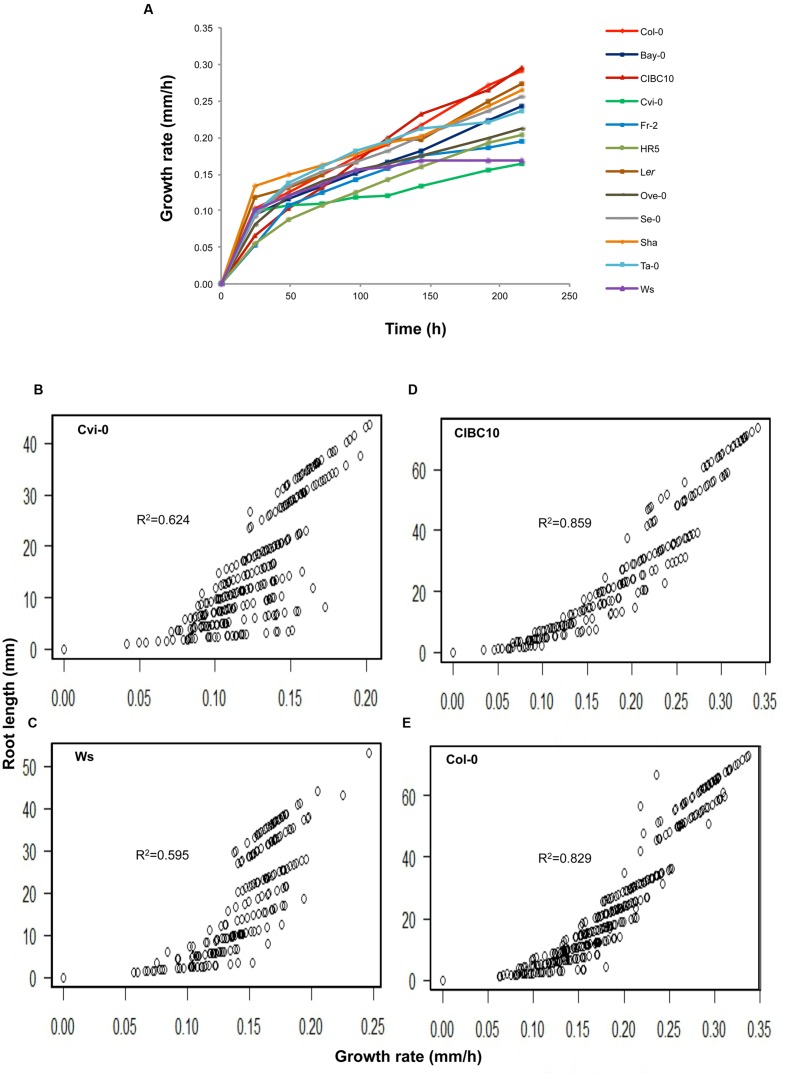
**Root growth rate variation among *Arabidopsis* accessions. (A)** Growth rate in millimeter/hour (mm/h) of Col-0, Bay-0, CIBC10, Cvi-0, Fr-2, HR5, L*er*, Ove-0, Se-0, Sha, Ta-0 and Ws unto 216 h after germination (*n* > 30). Correlation plots between root length and growth rate for Cvi-0 **(B)**, Ws **(C)**, CIBC10 **(D)**, and Col-0 **(E)**. Lineal correlation (*R*^2^) at *P* value < 2.2 × 10^-16^ is indicated in each plot.

According to root length at 9 dag, we classified Cvi-0 and Ws as short root plants, whereas L*er*, Col-0 and CIBC10 as long root plants (**Figure 4**). We also found that the roots of the Fr-2, HR5 and Ove-0 were short but slightly longer than Cvi-0 and Ws, whereas the ones from Se-0 and Sha were long but slightly shorter than L*er*, Col-0 and CIBC10, while the roots of the remaining accessions were of intermediate length (**Figure 4**). Normally, the root length is associated with its growth rate; however, in the first 24 h after germination, the growth rate of some accessions is not correlated with their root length at 9 dag, such as shown for Cvi-0 and Ws, that had a growth rate of 0.1 ± 0.02 mmh^-1^, similar to Col-0 and L*er*, while the former two accessions have short roots (**Figure 5A**). In contrast, CIBC10 growth rate was 0.07 ± 0.02 mmh^-1^, like that of HR5 and Fr-2, which are slightly short root accessions (**Figure 5A**). These discrepancies were due to the fact that the growth rate of Cvi-0 and Ws, two short root plants, were faster only in the first 24 h, but after that, growth rate was slightly lower than the long root accessions, exhibiting a non-linear trend between root length and growth rate with *R*^2^= 0.62 and 0.59 (*P* < 2.2 × 10^-16^), respectively (**Figures 5B,C**). Whereas CIBC10 and Col-0, two long root accessions, grew at a constant rate as shown in **Figures 5D,E**, in which the root length is proportional to the growth rate with *R*^2^= 0.86 and 0.83 (*P* < 2.2 × 10^-16^), respectively.

**FIGURE 6:**
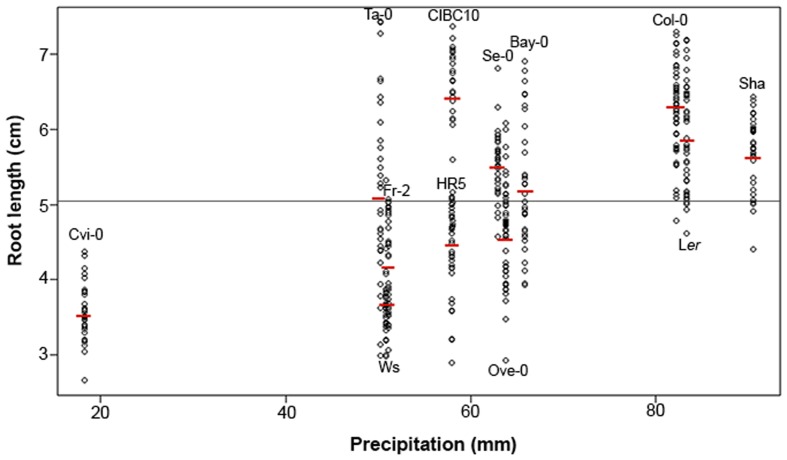
***Arabidopsis* root length variation and its relationship to precipitation.** Spearman correlation analysis between root length and the average annual precipitation from habitats of each *Arabidopsis* accession (*n* > 30), *r*_s_ = 0.60 at *P*–value <2.2 × 10^-16^. The red lines represent the root mean lengths for each accession. The gray line shows the mean root length of all *Arabidopsis* accessions (mean = 5.07).

Root growth and morphology depend partly on environmental conditions and these characteristics can frequently be retained and inherited in populations as an adaptive trade (Richards et al., 2012). To assess the relationship between root length and environmental conditions (**Table 1**), we obtained Spearman linear correlation coefficients to analyze the influence of temperature and precipitation in the root length. No correlation was found between root length and temperature (Supplementary Figure S2). However a positive Spearman rank correlation of *r*_s_= 0.60 (*P* < 2.2 × 10^-16^) was found between root length and precipitation (**Figure 6**), indicating that low levels of precipitation are associated with short root accessions and *vice versa*. This is well exemplified with Cvi-0, Sha and Col-0 accessions. Cvi-0 comes from Cape Verdi Island a habitat with lower levels of precipitation and the accession collected from here has a short root accession compared to Col (or the others), while Col-0 and Sha have long roots and come from Columbia and Pamiro-Alay, respectively, two habitats with high precipitations (**Figure 6**). Although not all accessions showed high correlations between their root length and precipitation conditions, these data indicate that annual precipitation levels, especially the most extreme conditions, are a more determinant factor than mean annual temperature in affecting root length in these *Arabidopsis* populations.

### *Arabidopsis* RAM Size Variation and Its Association with Root Length

**FIGURE 7:**
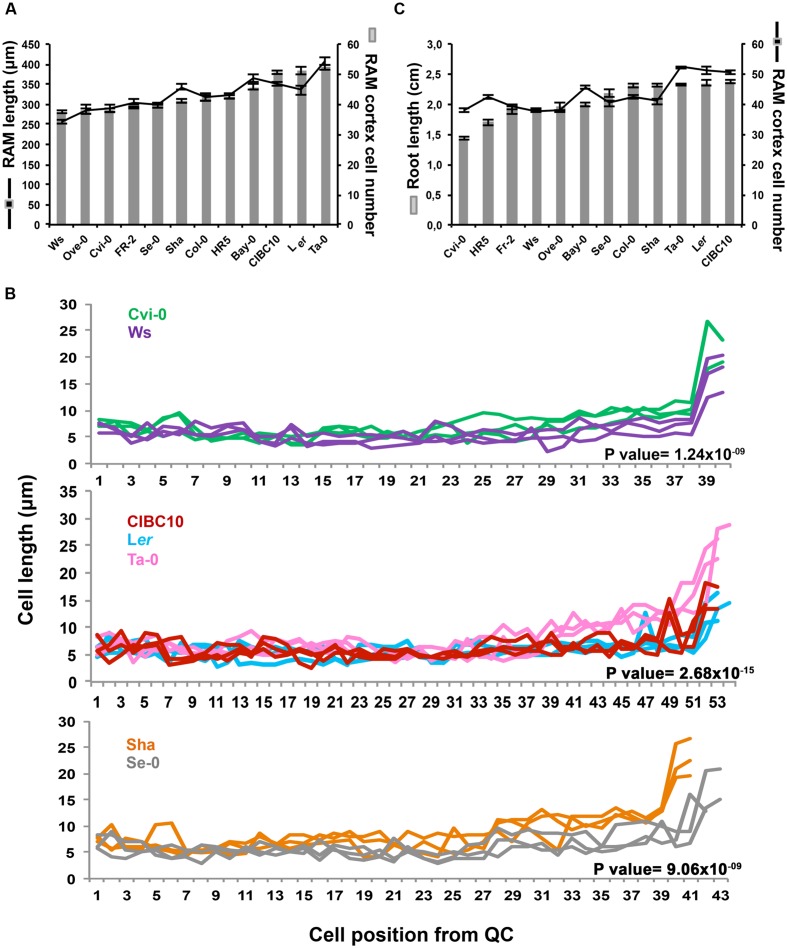
***Arabidopsis* root apical meristem variation and its association with root length. (A)** The RAM length in micrometers (μm) and RAM cortex cell number of Ws, Ove-0, Cvi-0, Fr-2, Se-0, Sha, Col-0, HR5, Bay-0, CIBC10, L*er*, and Ta-0; (*n* > 30). **(B)** Cell position from QC and the length of each cell from RAM of Cvi-0 (green), Ws (purple), CIBC10 (red), Ta-0 (pink), L*er* (light blue), Sha (orange) and Se-0 (gray). For better visualization, each plots only show a representative sample of *n* = 10. The *P*-value of two-way ANOVA analysis is shown on each plot and represents the differences in cell length as an effect of belonging to each accession **(C)** Root length in centimeters (cm) and RAM cortex cell number of *Arabidopsis* accessions; (*n* > 30). Seedlings of 5 dag were used. Bars represent standard error.

Root growth depends on the balance between cell proliferation and cell expansion, two processes associated with RAM homeostasis. Generally, a high proliferation rate at the RAM produces more cells that are able to differentiate and expand, resulting in a high growth rate (Baskin, 2013). To analyze the association of the RAM size with root length, we first evaluated the size of the RAM based on its length and the number of cortex cells from the QC to the first elongated cortex cell, separating small proliferative cells from large fast expanding cells, found in the elongation zone (Casamitjana-Martinez et al., 2003; Hacham et al., 2011). Interestingly, we found in some *Arabidopsis* accessions that the number of cortex cells in the RAM does not determine the RAM length. This was the case of Cvi-0 and Ws that share the same number of meristem cortex cells (37.67 ± 3.16) but their RAM length differs by 33.06 μm; likewise Sha and Se-0 as well as L*er*, CIBC10 and Ta-0, that contain 40.73 ± 4.53, and 51.30 ± 6.06 cortex cells and their RAM length differ 44.31 and 71.88 μm, respectively (**Figure 7A**). The differences in RAM length were due to variation in the size of cells close to elongation zone (the transition domain), as it is shown in Cvi-0, Ta-0 and Sha, which have larger cells than Ws, CIBC10 or L*er* and Se-0, respectively (**Figure 7B**). The statistic analysis in all comparisons indicate that the differences on cell length was significantly associated to the accession rather than its position (*P*-value Cvi-0/Ws = 1.24 × 10^-09^, *P* value CIBC10/L*er*/Ta-0 = 2.68 × 10^-15^, *P*-value Sha/Se-0 = 9.06 × 10^-09^), thus the differences between meristematic cell length in transition zone were due to intrinsic factors of each accession, and not to the position of the cells in the meristematic zone (*P*-value Cvi-0/Ws = 0.87, *P*-value CIBC10/L*er*/Ta-0 = 0.32, *P*-value Sha/Se-0 = 0.67) or to the interaction of both factors (*P*-value Cvi-0/Ws = 0.84, *P*-value CIBC10/L*er*/Ta-0 = 0.75, *P*-value Sha/Se-0 = 0.30). These data reveal that the discrepancies between RAM cortex cell number and length seem to be due to differences in the size of cells from the transition zone.

We then decided to evaluate the association between RAM and whole root length, considering only the cortex cell number. This was motivated by the fact that it has been reported that the number of cells in the RAM reflects its proliferative capacity, which has an important impact in root growth modulation (Baskin, 2013). Thus, RAM length was directly related with root length in Fr-2, Ws, Ove-0, and Se-0, as well as in Ta-0 and L*er* (**Figure 7C**), whereas the number of RAM cortex cells in Cvi-0 and HR5 were similar to Fr-2, Ws or Ove-0, however, their roots were shorter than the latter accessions, and similar behavior was observed in Bay (**Figure 7C**). We also found that Sha and Col-0, two long root accessions have a small RAM in comparison to L*er*, Ta-0 and CIBC10, which have largest RAM and long roots (**Figure 7C**). These results suggest that the number of RAM cortex cells is not always directly proportional to root length. In some *Arabidopsis* accessions other events occurring at the differentiation zone may be more relevant in determining root length. On the other hand, it is important to note that at this time (5 dag), the comparative analysis in root length among accessions differs from the results of roots at 9 dag (**Figure 4**). This is mainly so, in Ws and Ta-0, which at 9 dag have short and intermediate roots, respectively; whereas at 5 dag their length was intermediate for Ws and long for Ta-0. This provides additional evidence concerning the impact of a non-constant root growth rate.

### Root Stem Cell Niche Structure Variations among *Arabidopsis* Accessions

**FIGURE 8:**
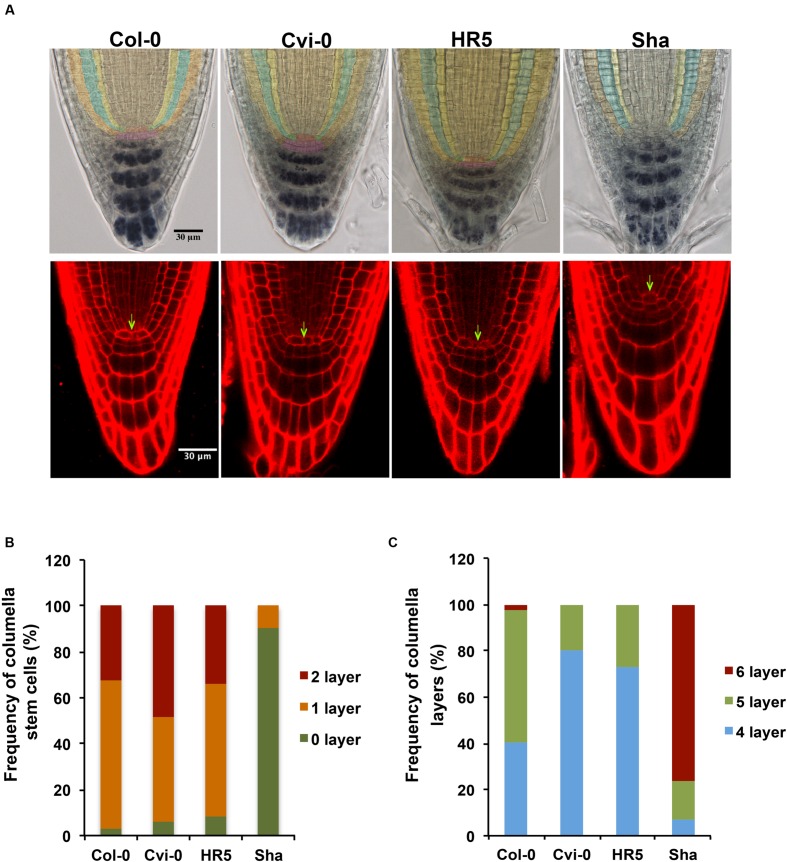
**Root stem cell niche morphology in different *Arabidopsis* accessions. (A)** Root stem cell niche morphology of Col-0, Cvi-0, HR5 and Sha. Lugol or PI staining of 4 dag roots. The QC cells are in red or indicated with arrows, cortex/endodermis stem cells are in green, columella stem cells are in pink, epidermal/lateral root cap stem cells are in brown, mature epidermal cells are in orange, cortex cells in blue and endodermis cells in yellow **(B)** Frequency of columella stem cells and **(C)** columella layers in Col-0, Cvi-0, HR5 and Sha. (*n* > 35).

The root stem cell niche organization of *Arabidopsis* has been described for Col-0, L*er* and Ws accessions (Tapia-Lopez et al., 2008; Kornet and Scheres, 2009). As it can be seen in **Figure 8**, the QC cells are surrounded by one layer of initial/stem cells of cortex/endodermal, as well as of columella and epidermal/lateral root cap cells, that can differentiate into different cell types (Dolan et al., 1993). When the stem cell niche of the different *Arabidopsis* accessions was analyzed, we found that the stem cell niche of Bay-0, CIBC10, Fr-2, Ove-0, Se-0 and Ta-0 has a similar organization as that of Col-0, in which generally a single layer of columella stem cells immediately distal to the QC (65%) are observed. While 48% of Cvi-0 and 34% of HR5 roots contain two layers of undifferentiated cells; in addition 80% of Cvi-0 and 73% of HR5 roots contain four tiers of differentiated columella cells with their characteristic starch granules stained with lugol (purple cells), in contrast to Col-0 in which 57% contain five and only 40% contain four differentiated columella layers (**Figures 8B,C**). Interestingly, we found that the stem cell niche of Sha has an uncommon organization, since the QC and columella stem cells are not well defined probably because they have aberrant divisions (**Figure 8**). In 90% of plants from the Sha accession we were unable to defined the columella stem cells, but interestingly, we could still find six layers of columella cells in 76% of the roots analyzed. These analyses indicated that the main natural variation in stem cell niche organization is in the columella stem and differentiated cells, however, molecular analysis will be necessary to determine the impact of these stem cell variations in root development.

## Discussion

The plant root is essential to provide nutrients, minerals and water, as well as to anchor the plant to the ground. The root system plastically adjusts its architecture depending on nutrient and water availability and also on soil structure (Aiken and Smucker, 1996). Although several genes have been identified to be involved in root growth, architecture and development (Slovak et al., 2015), we still have scarce information on the complex networks underlying variation in root architecture and growth, and the role of particular environmental factors in such variation and its adaptive evolution, as well as the role of plasticity.

This work shows that root architecture and meristem traits widely vary among 12 *Arabidopsis* accessions uncovering variation in: root architecture, root length, RAM size, as well as the structure of the stem cell niche. The atypical root architecture of HR5 with very few and short lateral roots, impeded its morphometric analysis. Hence, further studies should be pursued to evaluate how plastic is such extreme root phenotype and what gene alterations might be linked to it (Mouchel et al., 2004). On the other hand, the root architecture of Ws and Cvi-0 were the most contrasting ones showing a slanting root, that could affect lateral root orientation, to compensate contact area important for nutrient and moisture access (Ristova and Busch, 2014). Among them, Cvi-0 had higher procrustes distances in all pairwise comparisons and was the most distal in the multivariate space, these differences could be as a result of its adaptation to specific warm and dry climate (Lobin, 1983), which could give its contrasting response strategies to different environments (Rao and Davis, 1999; Borsani et al., 2001; Cooley et al., 2001; Aguilar et al., 2002).

We also found that root length does not affect root morphology, since only 7.26% of root morphology variation being attributable to root length variation. Root length depends on its growth rate (Beemster and Baskin, 1998), and our results revealed that growth rate is constant in some accessions, while in others it varies with age, like in Ws and Ta-0. Such variation in root growth rates may underlie the different root lengths observed among *Arabidopsis* accessions.

Previous studies have linked phenotypic variation to particular environmental conditions (Villellas et al., 2014). Root might grow slower to conserve nutrients or faster to reach new ones (Baskin, 2013). Interestingly, we found a positive significant correlation between root length and precipitation conditions of the different habitats from which accessions were collected. Our data indicate that low precipitation is associated with shorter root accessions and high levels of precipitation are related with longer root accessions. The Cvi-0 accession is the best example in which low levels of precipitation are associated with short root accessions. This contrasts with previous reports in which drought conditions were reported to induce primary root growth and inhibit lateral root development (Chaves et al., 2002; Smith and De Smet, 2012), although it is also known that some adverse conditions can inhibit root growth including water deficit stress (West et al., 2004; Yamaguchi et al., 2010; Baskin, 2013). Therefore other factors could be affecting the root behavior, in the case of Cvi-0, low humidity increases stomatal opening and the ABA levels (Monda et al., 2011), and even it loses water more rapidly in normal and drought conditions (Bouchabke et al., 2008). Although these Cvi-0 traits are from aerial tissues could be affecting root growth. Moreover root growth can also be affected in response to particular environmental conditions, as alterations in pH or the type of soil under such conditions (Smith and De Smet, 2012). For example, Sha accession was collected from Tajikistan, Central Asia near to the Shokhdara river that irrigate the valley during summer when precipitation is limited and coinciding with flowering time (Trontin et al., 2011), this particular environment could be contributing differentially at Sha root phenotype traits. Future studies should investigate to know if the accessions root growth is plastic under contrasting soil humidity conditions, or if these traits are fixed.

Stem cell division rate and cell proliferation rates at the RAM, as well as differentiation rate and cell elongation are intrinsic processes that also alter root length (Ivanov and Dubrovsky, 2013). Our data showed that RAM is not always an indicator of the number of dividing cells, as it has been reported for Col-0 accession (Beemster and Baskin, 1998; Shishkova et al., 2008). The discrepancies found between RAM length and RAM cell number in Ws, Sha, CIBC10 and L*er* are rather explained by cell length at the transition domain. On the other hand, number of cell in the RAM reflects its proliferative capacity and alters root length, with high proliferation rate at the RAM producing more cells able that eventually differentiate, and increase root growth rate (Baskin, 2013). But such relationship is not applicable to all accessions studied. Some of these had short roots with a relatively higher number of cells at the RAM in comparison to other accessions, which had fewer cells at the RAM, but yielded longer roots. These results indicate that root growth patterns vary among accessions with respect to those described in Col-0. In some accessions, cell elongation seems to be more relevant to explain total root length rather than meristem length (Beemster et al., 2002).

The stem cell niche organization and behavior has a direct impact on RAM homeostasis and also influences root architecture (Shishkova et al., 2008). Columella stem cells and the number of columella differentiated layers showed the greatest range of variation among accessions. Interestingly, the extra layer of undifferentiated stem cell found in Cvi-0 and HR5 are reminiscent of the loss-of-function mutant in the *RETINOBLASTOMA-RELATED* (*RBR*) gene, which is defective on promoting stem cell differentiation (Wildwater et al., 2005). In contrast, the undefined QC cells and columella stem cells, as well as the extra columella cells found in Sha, suggest an accelerated differentiation of columella stem cells, similar to the behavior of loss of QC identity by QC ablation or by the loss-of-function mutants in the *WUSCHEL-RELATED HOMEOBOX 5* (*WOX5*) gene, which is normally expressed at the QC and is required to maintain stem cell identity (Wildwater et al., 2005; Sarkar et al., 2007). The accelerated differentiation in Sha could explain the discrepancies between its short meristem and long root, in where RAM cells are differentiated faster, generating more differentiated cells able to elongate and produce plants with long roots and also with more lateral roots. Therefore in this case the RAM size is less relevant than elongation or maturation zone in the determination of root length.

The uncovered root phenotypes among *Arabidopsis* accessions may be used to study the role of gene regulatory networks involved in such phenotypes. These data also open the door to studies of plasticity and its role in adaptive evolution (Espinosa-Soto et al., 2011) or the constructive role of different environmental factors, once contrasting growth conditions is tested for each accession.

## Author Contributions

PA-G and MP performed the experiments and data evaluation; MP designed, supervised and coordinated the experiments; AG-A, BG-P and EA-B participated in data interpretation and also discussed and performed a critical revision of the article; RM contributed with statistical analyses. PA-G, MP, AG-A, BG-P, and EA-B wrote the manuscript. All authors read and approved the final manuscript.

## Conflict of Interest Statement

The authors declare that the research was conducted in the absence of any commercial or financial relationships that could be construed as a potential conflict of interest.
