# Supplementary material for: Root Architecture Diversity and Meristem Dynamics in Different Populations of Arabidopsis thaliana
Source: Front Plant Sci. 2016 Jun 16;7:858. doi: 10.3389/fpls.2016.00858 (PMC4910468; doi:10.3389/fpls.2016.00858)
Supplement: Supplementary file 1 [file Image_1.PDF]

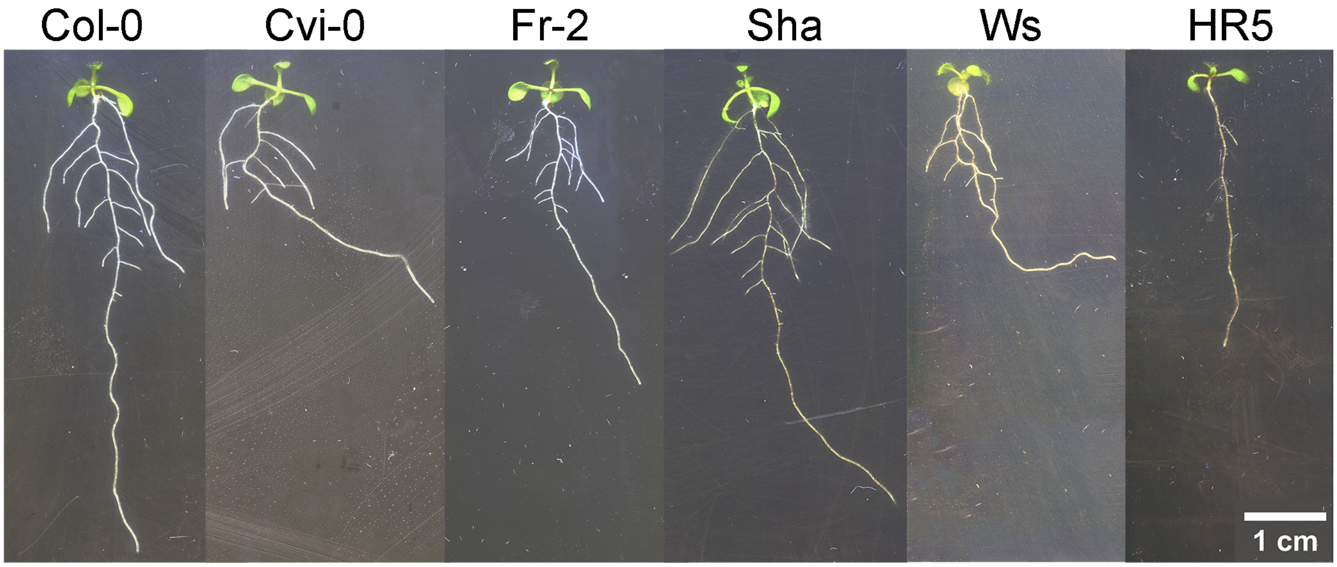

Supplementary Figure 1. Root morphology of *Arabidopsis* accessions. Root morphology of Col-0, Cvi-0, Fr-2, Sha, Ws and HR5 at 9 dag.

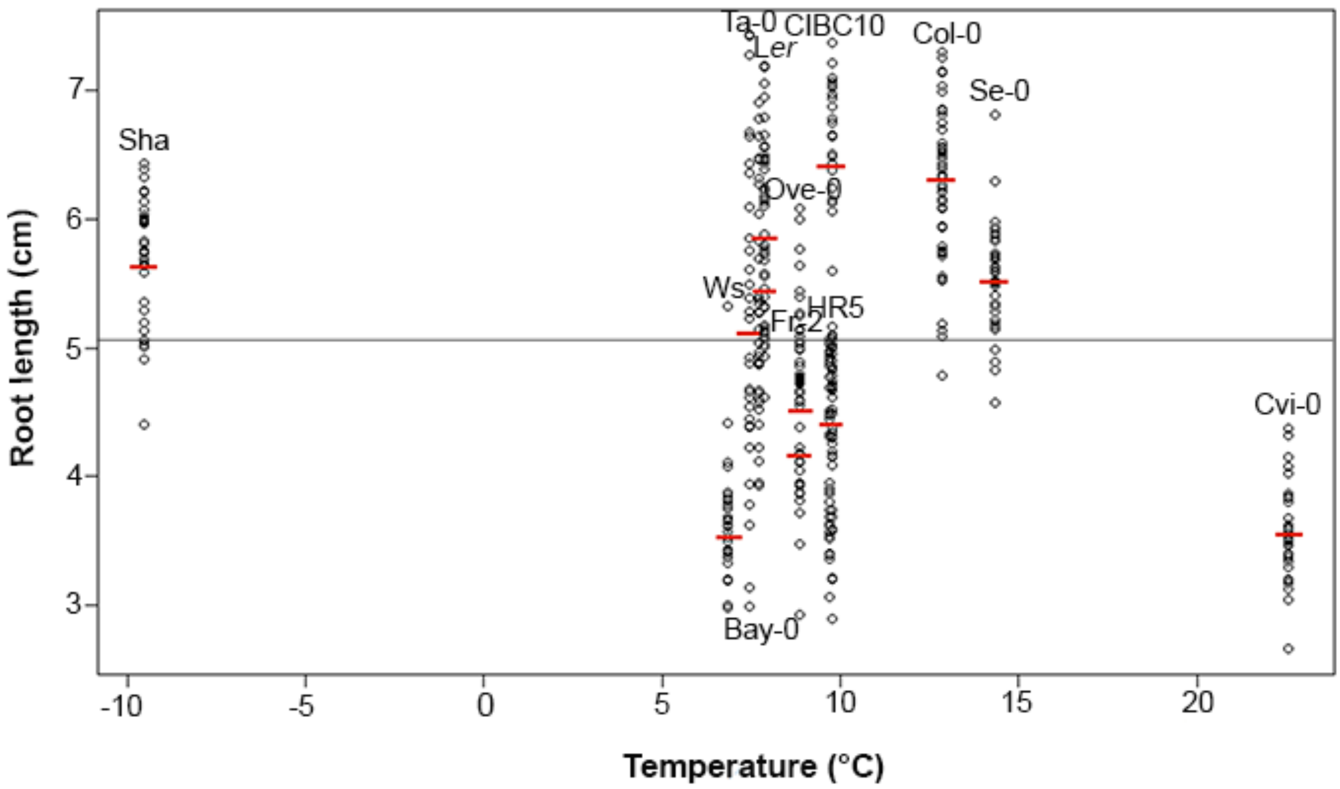

Supplementary Figure 2. *Arabidopsis* root length variation and its relation with temperature conditions. Spearman correlation analysis between root length and average annual temperature from habitats of each *Arabidopsis* accession (n>30). r_s= 0.022. at p value<$2.2X10^{-16}$. The red lines represent the root mean lengths for each accession. Gray line shows the mean root length of all *Arabidopsis* accessions (mean=5.07).

	Bay-0	ClBC10	Col-0	Cvi-0	Fr-2	L*er*	Ove-0	Se-0	Sha	Ta-0	Ws
**Bay-0**	0										
**ClBC10**	0.12	0									
**Col-0**	0.08	0.09	0								
**Cvi-0**	0.24	0.29	0.23	0							
**Fr-2**	0.08	0.11	0.05 *	0.21	0						
**L*er***	0.16	0.21	0.15	0.10	0.13	0					
**Ove-0**	0.09	0.14	0.11	0.30	0.12	0.23	0				
**Se-0**	0.08 *	0.06 *	0.05 *	0.26	0.07	0.17	0.11	0			
**Sha**	0.08 *	0.14	0.08	0.21	0.09	0.14	0.10	0.11	0		
**Ta-0**	0.08 *	0.16	0.09	0.26	0.10	0.19	0.07 *	0.11	0.08	0	
**Ws**	0.13	0.23	0.15	0.15	0.15	0.12	0.18	0.19	0.11	0.14	0

Supplementary Table 1. Comparative analysis of *Arabidopsis* root shape variation. Procrustes distances of pairwise comparison between all *Arabidopsis* accessions (n>10). Statistical significance at $p<0.05$. (*) indicates no significant differences.
